# Gut Microbial Gene Expression in Mother-Fed and Formula-Fed Piglets

**DOI:** 10.1371/journal.pone.0012459

**Published:** 2010-08-27

**Authors:** Valeriy Poroyko, James Robert White, Mei Wang, Sharon Donovan, John Alverdy, Donald C. Liu, Michael J. Morowitz

**Affiliations:** 1 Department of Surgery, The University of Chicago Pritzker School of Medicine, Chicago, Illinois, United States of America; 2 Department of Pediatrics, The University of Chicago Pritzker School of Medicine, Chicago, Illinois, United States of America; 3 Center for Bioinformatics and Computational Biology, University of Maryland, College Park, Maryland, United States of America; 4 Division of Nutritional Sciences, University of Illinois, Urbana-Champaign, Illinois, United States of America; Charité-Universitätsmedizin Berlin, Germany

## Abstract

**Background:**

Effects of diet on the structure and function of gut microbial communities in newborn infants are poorly understood. High-resolution molecular studies are needed to definitively ascertain whether gut microbial communities are distinct in milk-fed and formula-fed infants.

**Methodology/Principal Findings:**

Pyrosequencing-based whole transcriptome shotgun sequencing (RNA-seq) was used to evaluate community wide gut microbial gene expression in 21 day old neonatal piglets fed either with sow's milk (mother fed, MF; n = 4) or with artificial formula (formula fed, FF; n = 4). Microbial DNA and RNA were harvested from cecal contents for each animal. cDNA libraries and 16S rDNA amplicons were sequenced on the Roche 454 GS-FLX Titanium system. Communities were similar at the level of phylum but were dissimilar at the level of genus; *Prevotella* was the dominant genus within MF samples and *Bacteroides* was most abundant within FF samples. Screened cDNA sequences were assigned functional annotations by the MG-RAST annotation pipeline and based upon best-BLASTX-hits to the NCBI COG database. Patterns of gene expression were very similar in MF and FF animals. All samples were enriched with transcripts encoding enzymes for carbohydrate and protein metabolism, as well as proteins involved in stress response, binding to host epithelium, and lipopolysaccharide metabolism. Carbohydrate utilization transcripts were generally similar in both groups. The abundance of enzymes involved in several pathways related to amino acid metabolism (e.g., arginine metabolism) and oxidative stress response differed in MF and FF animals.

**Conclusions/Significance:**

Abundant transcripts identified in this study likely contribute to a core microbial metatranscriptome in the distal intestine. Although microbial community gene expression was generally similar in the cecal contents of MF and FF neonatal piglets, several differentially abundant gene clusters were identified. Further investigations of gut microbial gene expression will contribute to a better understanding of normal and abnormal enteric microbiology in animals and humans.

## Introduction

The principles that govern early microbial colonization in the mammalian intestine are poorly understood. Environmental exposures such as early infant diet are believed to impact the development and function of gut microbial consortia [1]. However, attempts to identify similarities and differences among gut microbes from breast-fed and formula-fed infants have generated conflicting and controversial results. For decades, culture-based studies of enteric microbiology supported the notion that the benefits of breast milk derive partly from the growth of beneficial probiotic bacteria in milk fed infants [1], [2]. Recently, this idea has been called into question by studies that used culture-independent methodologies [3]–[6]. Nonetheless, it is well accepted that some microbe-mediated diseases including neonatal necrotizing enterocolitis (NEC) are less common in mother-fed (MF) infants than in formula-fed (FF) infants [7], [8]. For this reason, high-resolution studies are needed to better characterize both the fine-scale structure and function of gut microbial communities in MF and FF neonates.

Cultivation-independent techniques have made it possible to identify many or most of the gut microbes present within biological specimens such as fecal samples or gut mucosal biopsies [9], [10]. However, partly due to the complexity of gut microbial consortia, little is known about which microbial genes and proteins are expressed *in vivo* within the gastrointestinal tract. To date, functional analysis of gut microbes has largely been limited to predictions of functional potential based upon DNA sequence annotations, e.g. with metagenomic analyses [9], [11]. While a handful of studies have used unbiased shotgun approaches to measure gut microbial proteins [12], [13] and metabolites [14], [15], only two recent studies [16], [17] have begun the essential work of characterizing community wide gene expression (the metatranscriptome) in the GI tract, as has been accomplished in studies of marine [18]–[21] and soil microbes [22], [23]. High-throughput investigations of gene expression make it possible to study how the phenotypes of natural microbial communities change over time in response to environmental exposures.

RNA-sequencing (RNA-seq) [24], [25] is a promising experimental approach to profiling gene expression with high-throughput sequencing technologies. Also referred to as whole transcriptome shotgun sequencing, this technique involves directly sequencing cDNA libraries to learn about the RNA content within a biologic sample. In contrast to most array-based techniques, RNA-seq allows for the characterization of both known and unknown gene transcripts. Furthermore, data output in the form of discrete nucleotide sequences rather than hybridization signals makes it possible to compare results from different laboratories and to study fine-scale variations in transcript sequences. Originally described in studies of eukaryotic gene expression, RNA-seq has now been used to profile gene expression in microbial isolates [26] and also within natural mixed population communities of the ocean and soil [18]–[23]. This approach holds particular promise for studying the function of complex gut microbial communities without knowing *a priori* which organisms are present.

Here, an unbiased RNA-Seq approach utilizing massively parallel pyrosequencing was used to study microbial gene expression in cecal contents from 21-day-old mother-fed (MF) and formula-fed (FF) neonatal piglets. Because nutrient availability is tightly linked to global transcriptional control and upregulation of bacterial virulence programs [27], [28], we hypothesized that patterns of gene expression would be distinct in MF and FF animals. To test this hypothesis, four piglets receiving sow's milk and four piglets receiving an artificial piglet formula were sacrificed at 21 days of life. Both DNA and RNA from the cecal contents of each animal were harvested and cryopreserved at the time of sacrifice. 16S ribosomal RNA gene sequences within the DNA libraries were amplified, sequenced, and characterized. In parallel, we isolated RNA from each sample and constructed corresponding whole genome cDNA libraries. The cDNA libraries were subsequently pyrosequenced and, where possible, annotated. Differentially abundant features between the MF and FF animals were identified with Metastats, a recently developed statistical method for comparing high-throughput sequencing data in discrete treatment populations on the basis of count data [29].

## Results

### DNA and cDNA-based Community Profiling

Two parallel approaches were used to identify the microbial organisms present within cecal contents from the 8 piglets studied. We amplified and sequenced 16S ribosomal DNA sequences from the DNA samples, and we also characterized unamplified fragments of 16S rRNA sequences present within the whole genome cDNA libraries (Table 1).

**Table 1:** Sequencing statistics of DNA and cDNA libraries from mother-fed and formula-fed piglets.

	Mother-Fed				Formula-Fed					
	MF1	MF2	MF3	MF4	FF1	FF2	FF3	FF4	Total	Average
Total cDNA sequences	108,960	87,406	87,257	85,377	93,935	93,073	97,970	113,446	767,424	95,928
Average read length (bp)	376	384	373	376	367	374	352	339	n.a.	367
Total library size (Mb)	41	33.6	32.5	32.1	34.5	34.8	34.4	38.4	281.3	35.2
Total number of sequences <100 bp	6606	4820	5757	5393	5891	5563	6456	8361	48847	6106
Total rRNA sequences screened	70,724	62,765	55,630	58,592	70,178	81,279	83,962	96,333	579,463	72,433
Total non-ribosomal RNA sequences	31,630	19,821	25,870	21,392	17,866	6,231	7,552	8,752	139,114	17,389
% of non ribosomal RNA sequences	30.90	24.00	31.74	26.75	20.29	7.12	8.25	8.33	n.a.	19.67
Total size of non-rRNA library (Mb)	11.89	7.61	9.65	8.04	6.56	2.33	2.66	2.97	51.71	6.46
Total sequences matched to proteins in SEED subsystems	6,208	7,097	4,007	3,017	9,199	2,284	2,858	2,430	37,100	4,638
% total sequences assigned to SEED subsystems	5.7	8.1	4.6	3.5	9.8	2.5	2.9	2.1	n.a.	4.8
% non rRNA sequences assigned to SEED subsystems	19.6	35.8	25.5	14.1	51.5	36.7	37.8	27.8	n.a.	31.1
Total sequences matched to COG database	6,361	7,140	4,068	3,044	8,708	2,382	2,827	2,507	37,037	4,630
% total sequences assigned to COG	5.84	8.17	4.66	3.57	9.27	2.56	2.89	2.21	n.a.	4.90
% non rRNA sequences assigned to COGs	20.11	36.02	15.72	14.23	48.74	38.23	37.43	28.64	n.a.	29.90
Total non-ribosomal sequences annotated to level of phylum	4,824	5,266	3,236	2,172	10,079	2,002	2,518	2,864	32,961	4,120
% non rRNA sequences annotated to level of phylum	15.3	26.6	12.5	10.2	56.4	32.1	33.3	32.7	n.a.	27.4
Total 16S rDNA amplicon sequences >100 bp	756	760	990	634	860	578	767	734	6,079	760
Average amplicon sequence length (bp)	437.7	431.8	446.6	445.1	413.7	415.6	458.8	469.3	n.a.	439.8

**Figure 1:**
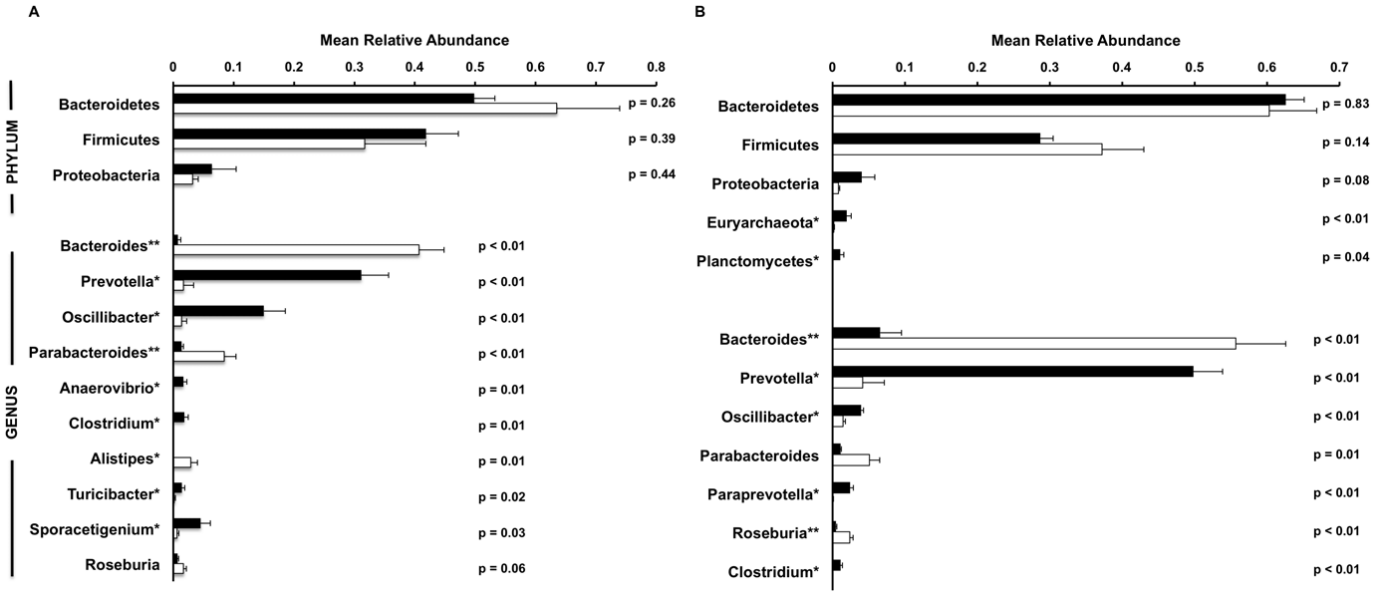
Microbial community structure in cecal contents from mother-fed and formula-fed piglets at 21 days of life. (A) Mean relative abundances of bacterial taxa within cecal microbiota from 4 MF animals (*black bars*) and 4 FF animals (*white bars*) identified by analysis of 16S rDNA amplicon sequences. Sequences were classified to the highest taxonomic level to which they could be confidently assigned using the RDP classification algorithm and taxonomic hierarchy. (B) Relative mean abundances of bacterial taxa within cecal microbiota identified by RDP analysis of unamplified 16S sequences within cDNA libraries. The results demonstrate that all communities are dominated by the phyla Bacteroidetes and Firmicutes; MF data sets are enriched with *Prevotella* sequences and FF data sets are enriched with *Bacteroides* sequences. In both panels, differentially abundant phyla (p value<0.05) and genera (p value<0.01) are marked with a * if they were enriched within the MF samples and with a ** if they were enriched within FF samples. Taxa with a mean relative abundance <0.01 in both MF and FF groups are not shown.

Analysis of 16S rDNA amplicons demonstrated the presence of microbes from 11 total phyla. However, 98% of 16S rDNA amplicons classified at the level of phylum were derived from the phyla Bacteroidetes (56.8% of total), Firmicutes (36.6%), and Proteobacteria (4.8%). At the level of phylum, no taxa were differentially abundant within either the MF or FF group. However, several genera were differentially abundant in one of the two animal groups (Figure 1a and Table S1). The MF data sets were enriched (p<0.01) with sequences attributed to the genera *Prevotella*, *Oscillibacter*, and *Clostridium*; the FF data sets were enriched (p<0.01) with sequences from *Bacteroides*, *Parabacteroides*, and *Alistipes*. *Prevotella* represented the most abundant genus in MF animals but was nearly absent in FF animals. Conversely, *Bacteroides* was the most abundant genus in FF animals but was largely absent in MF animals. The genus *Lactobacillus* was relatively enriched within MF samples (MF mean relative abundance 0.09 vs FF mean relative abundance 0.02), although this discrepancy did not reach statistical significance (p = 0.13). *Bifidobacterium* sequences were not observed in any sample. Principal component analysis of this data set (Figure S1) demonstrated that samples from the MF and FF groups cluster separately at the level of genus.

The sequenced cDNA libraries (after one cycle of mRNA enrichment) contained 80.6% ribosomal RNA sequences, and taxonomic assignments were successfully made for 4.0% of these sequences (25,398 total sequences) using the Ribosomal Database Project classification algorithm [30] (Figure 1B). Assessment of microbial community structure with this approach was quite similar, particularly for abundant organisms, to the assessments made by analyzing PCR amplicons of 16S rDNA sequences. Both analyses revealed a similar distribution of sequences from the three major phyla; further, both analyses demonstrate the enrichment of *Prevotella*, *Oscillibacter*, and *Clostridia* sequences in the MF samples and the enrichment of *Bacteroides* sequences in the FF samples. The relative agreement of results in Figure 1A and Figure 1B indicate that RNA-seq of total RNA isolated from mixed population consortia is an accurate means of determining community structure in the GI tract; as a prior study also indicated (23), analysis of 16S rDNA amplicons may not be required in this setting.

### A core microbial metatranscriptome in the piglet cecum

While increasing attention has been paid recently to “core” indispensable genes and organisms that are present within mammalian gut microbial communities [9], [31], [32], little is known about how the expression of these genes varies in response to environmental conditions. To address this issue, barcoded cDNA libraries were constructed from microbial RNA extracted from the cecal contents of all 8 animals after performing one cycle of mRNA enrichment. Pyrosequencing of these libraries yielded 767,424 total reads with an average read length of 381 bp (281.3 Mb total sequence) (Table 1). Automated removal of ribosomal RNA sequences yielded 98,713 and 40,401 non-ribosomal RNA sequences from MF and FF animals, respectively (51.7 Mb total). Screened cDNA sequences were assigned functional annotations by MG-RAST [33] and additionally based upon best-BLASTX-hits to the NCBI COG database [34] (e-values<1e^−5^ for both methods of annotation). Taxonomic assignments for cDNA sequences were also made where possible.

**Figure 2:**
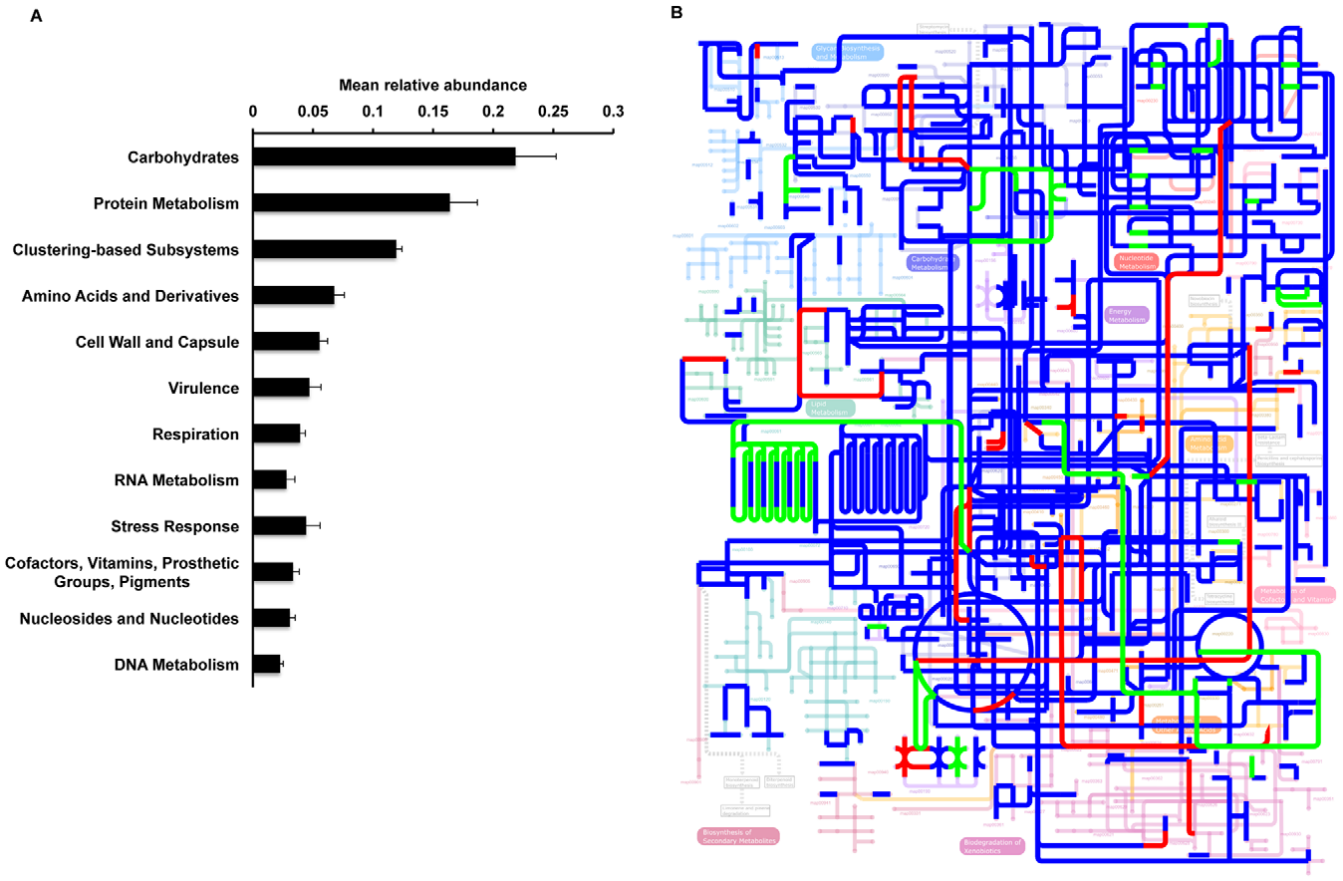
A core microbial metatranscriptome in the piglet cecum. (A) Mean relative abundances of annotated sequences within cDNA libraries from all 8 animals studied. Displayed are the automated SEED Level 1 Subsystem assignments, as determined by MG-RAST [33]. Low standard deviations indicate that variation in the gut metatranscriptome between subjects is low. (B) Projection of the global metabolic profiles onto the KEGG pathways using the iPath tool [35] demonstrates the overall similarity of the MF and FF gut microbial communities. Metabolic pathways common to both diets are shown in *blue*. Pathways unique to the MF animals are represented in *green*, and pathways unique to the FF animals are represented in *red*.

Consistent with other studies of gene expression within marine and soil microbial consortia [18], [20]–[23], most transcripts could not be annotated. 31% of non-ribosomal RNA sequences could be matched to proteins in the SEED Subsystems database, and 30% of sequences were matched to the COG database (e-value cutoff of 1e^−5^ for both analyses) (Table 1). Similar to published metagenomic and metatranscriptomic studies of terrestrial and environmental microbial communities, over 30% of annotated transcripts were associated with either carbohydrate or protein metabolism (Figure 2a and Table 2). Sequences linked to amino acid metabolism, stress response, respiration, virulence, and cell wall metabolism were also well represented. Figure 2 demonstrates that the mean abundance of transcripts within MG-RAST subsystems was remarkably constant across all 8 animals; for all subsystems, the average standard deviation of the mean relative abundance from all animals was 1.0%. Projection of the global metabolic profiles onto the KEGG pathways using the iPath tool [35] further demonstrated the overall similarity of the MF and FF the gut microbial communities (Figure 2b).

**Table 2:** Annotations of highly expressed microbial genes in the piglet cecum.

SEED Annotations			COG hits			
Rank	SEED Level 3 Subsystem	Raw number of hits	Rank	COG	Annotation	Raw number of hits
1	Ribosome_LSU_bacterial	1725	1	COG0057	Glyceraldehyde-3-phosphate dehydrogenase/erythrose-4-phosphate dehydrogenase	620
2	Ribosome_SSU_bacterial	1226	2	COG0050	GTPases - translation elongation factors	593
3	Universal_GTPases	1149	3	COG1592	Rubrerythrin	544
4	Oxidative_stress	943	4	COG0480	Translation elongation factors (GTPases)	525
5	Pyruvate_metabolism_I:_anaplerotic_reactions__PEP	814	5	COG0662	Mannose-6-phosphate isomerase	368
6	tRNA_aminoacylation	786	6	COG0448	ADP-glucose pyrophosphorylase	306
7	Sialic_Acid_Metabolism	778	7	COG0574	Phosphoenolpyruvate synthase/pyruvate phosphate dikinase	302
8	Entner-Doudoroff_Pathway	673	8	COG0334	Glutamate dehydrogenase/leucine dehydrogenase	302
9	Maltose_and_Maltodextrin_Utilization	672	9	COG0191	Fructose/tagatose bisphosphate aldolase	290
10	Mannose_Metabolism	621	10	COG0542	ATPases with chaperone activity, ATP-binding subunit	280
11	Ton_and_Tol_transport_systems	621	11	COG1653	ABC-type sugar transport system, periplasmic component	236
12	Sucrose_Metabolism	600	12	COG0674	Pyruvate∶ferredoxin oxidoreductase and related 2-oxoacid∶ferredoxin oxidoreductases, alpha subunit	232
13	Ribosome_activity_modulation	593	13	COG0443	Molecular chaperone	229
14	Lactose_and_Galactose_Uptake_and_Utilization	564	14	COG1544	Ribosome-associated protein Y (PSrp-1)	227
15	Pyridoxin_(Vitamin_B6)_Biosynthesis	548	15	COG1109	Phosphomannomutase	214

The table lists the most heavily represented SEED Level 3 Subsystems (*left* aspect of table) and the most abundant hits to the Clusters of Orthologous Groups (COGs) database (*right* aspect of table). Total number of hits reflects sum total of all hits across all 8 study subjects (i.e. both MF and FF animals).

**Figure 3:**
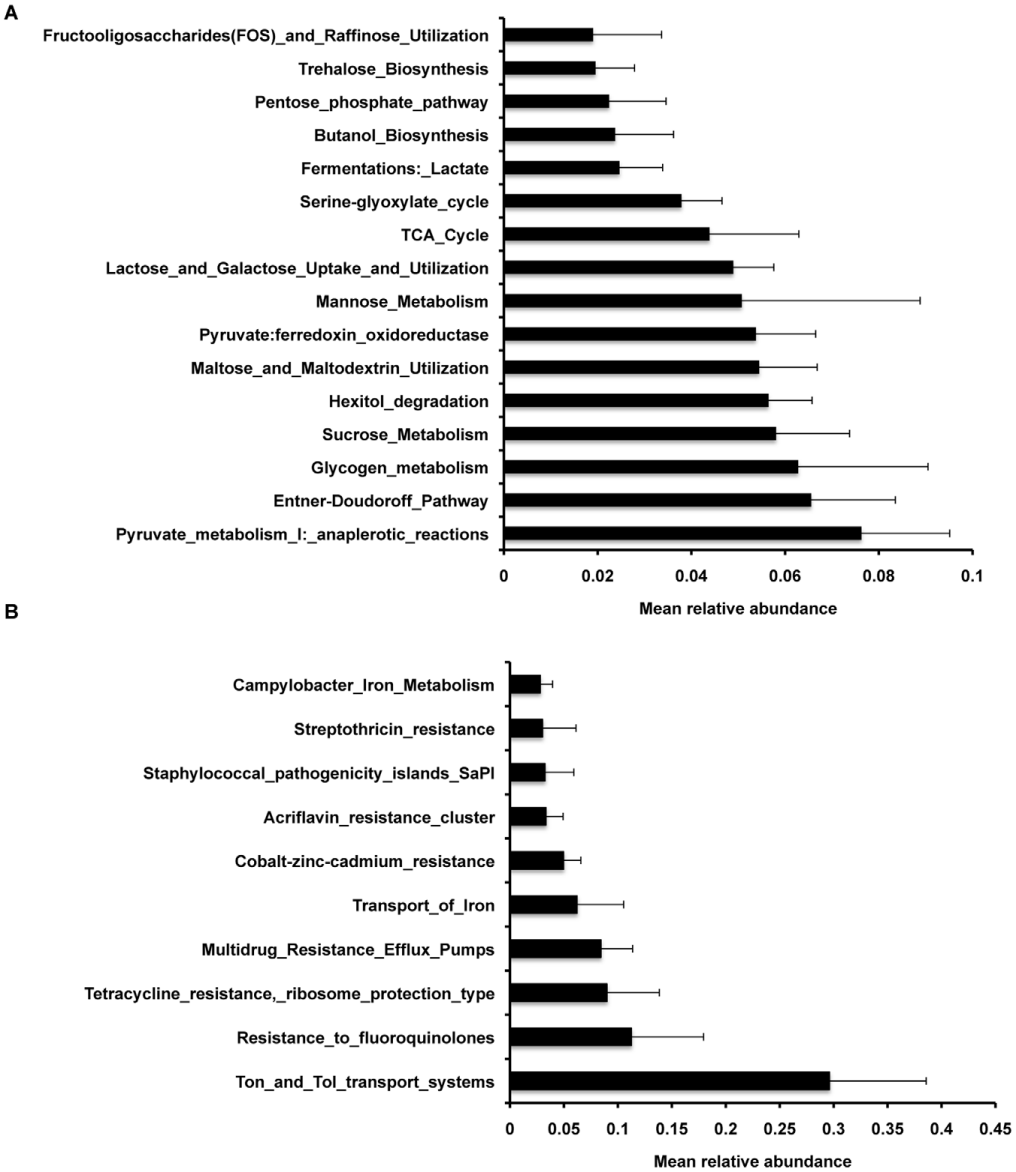
Universal expression of carbohydrate utilization and microbial virulence genes in the piglet cecum. (A) Relative abundance of cDNA sequences assigned by MG-RAST to the Level 3 SEED Subsystem of carbohydrate utilization. (B) Relative abundance of cDNA sequences assigned by MG-RAST to the Level 3 SEED Subsystem of virulence. Values for mean relative abundances in both (A) and (B) reflect average values across all 8 animals studied.

Carbohydrate utilization profiles (Figure 3a) were dominated by transcripts linked to central carbohydrate and glycogen metabolism subsystems. The single most abundant COG hit was to glyceraldehyde-3-phosphate dehydrogenase (GAPDH; COG0057); other common transcripts encoded pyruvate phosphate dikinase (COG0574), phosphoglycerate kinase (COG0126), and glycogen synthase (COG0297) (Table 2). Sequences linked to utilization of mono- and oligosaccharides were also abundant, and were assigned to SEED subsystems for metabolism of sucrose, maltose, lactose, fructooligosaccharides, and sugar alcohols (hexitol). These sequences included transcripts for mannose-6-phosphate isomerase (COG0662), phosphomannomutase (COG1109), beta-galactosidase (COG3250; lactose utilization), and tagatose-bisphosphate aldolase (COG0191; galactose utilization). Sequences associated with ABC-type sugar transporters (COG1653, COG1879, COG3839, COG0395, COG1175, COG1129) and sugar-specific phosphotransferases (COG3444, COG3716, COG2893, COG1263) were commonly observed.

Transcripts associated with virulence, stress response, and cell wall metabolism were also relatively abundant across all animals (mean relative abundances 0.055±0.007, 0.047±0.01, and 0.044±0.01, respectively). The most abundant sequences classified within the SEED subsystem of virulence were associated with Ton and Tol transport systems, fluoroquinolone resistance, and other iron transport systems (Figure 3b). The third most commonly observed COG overall was rubrerythrin (COG1592), a protein that provides protection from oxidative stress via catalytic reduction of intracellular hydrogen peroxide [36]. Other oxidative stress response genes, such as peroxiredoxin (COG0450) and superoxide dismutase (COG0605), were also highly expressed. Abundant transcripts in the cell wall and capsule subsystem included numerous genes regulating sialic acid metabolism (COG4409, COG0363, COG0329, COG0774, COG1820, COG2942) and genes mediating utilization of plant-based polysaccharides via the cellulosome (e.g. SusC, which encodes an outer membrane protein involved in starch binding in *Bacteroides* species [37]).

Several of the commonly expressed microbial genes in the piglet cecum have crossover functions linking their primary function with binding to host epithelium, lipopolysaccharide (LPS) metabolism, oxidative stress response, and virulence. GAPDH, beta-galactosidase, enolase (COG0148; central carbohydrate metabolism), phosphoglycerate mutase (COG0588; central carbohydrate metabolism) and elongation factor Tu (COG 0050) have all been noted to mediate binding to host mucins, fibrinogen, and/or plasminogen [38]–[43]. Interestingly, it has been demonstrated that in vitro secretion of GAPDH by *E coli* varies according to the composition of nutrients in the growth medium [44]. Mannose-6-phosphate isomerase [45] and glutamate dehydrogenase [46] (GDH; COG0334) each have been shown to contribute to microbial survival in the face of oxidative stress. Mannose-6-phosphate isomerase, periplasmic ABC transporters, and phosphomannomutase are involved with processing of extracapsular polysaccharides such as LPS [45], [47]. Another commonly observed transcript, phosphoenolpyruvate phosphotransferase (COG1080) was recently demonstrated to jointly control carbohydrate utilization, biofilm formation, and intestinal colonization in a *Vibrio cholerae* model [48]. Finally, decreased pathogenicity has been observed in models with mutagenesis or inhibition of GAPDH and beta galactosidase [38], [49]. Because these genes were highly expressed in all samples, it is likely that these genes represent core features of microbial communities in the piglet cecum.

### Taxonomic assignments for cDNA sequences

**Figure 4:**
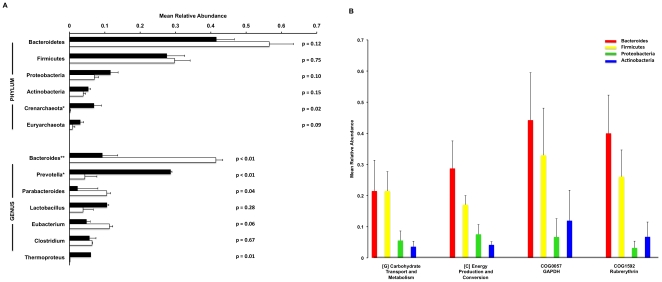
Relationship between gut microbial community structure and function. (A) Mean relative abundances of bacterial taxa within cecal microbiota from 4 MF animals (*black bars*) and 4 FF animals (*white bars*) identified by analysis of non-ribosomal sequences within cDNA libraries. Taxonomic assignments for sequenced transcripts were made by identifying best BLASTN hits against an in-house database of microbial genomes. Differentially abundant phyla (p value<0.05) and genera (p value<0.01) are marked with a * if they were enriched within the MF samples and with a ** if they were enriched within FF samples. (B) Taxonomic origin of highly expressed genes. Expression levels for the two most abundant general COG hits and two most abundant individual COG hits are represented for each of the four most abundant bacterial phyla.

An advantage of digital studies of the community transcriptome is that the data can inform explanations of how metabolic tasks are divided among the members of gut microbial communities. Little is currently known about whether fundamental processes such as carbohydrate utilization are performed by a range of organisms in vivo or rather by a limited group of specialized community members. Furthermore, although it is widely recognized that numerous low abundance bacterial species reside in the GI tract, little is known about the contribution of such organisms to community metabolism and function. To begin to address these questions, taxonomic assignments were made, where possible, for each gene transcript by identifying the best BLASTN hit against an in-house database of 1054 finished microbial genomes (Table S2). In both MF and FF groups, non-ribosomal transcripts from the phylum Bacteroidetes were most abundant (Figure 4a). In general, the abundance of transcripts from a given phylum was similar to the population wide abundance of that phylum as determined by the 16S- based methods (Figure 1). In Figure 4b, the two most abundant general COG and two most abundant individual COG hits are listed along with the taxa linked to the transcripts.

These data highlight the importance of available reference microbial genomes when attempting to profile mixed population microbial communities *without* knowing in advance which organisms are present. The taxonomic origin could be assigned for only 27.4% of all non-ribosomal sequences. In the MF group, which was dominated by organisms from the genus *Prevotella*, only 16% of sequences could be linked to a taxonomic origin (Table 1). Most likely, the gene content of the strains present in these samples differs substantially from the three *Prevotella* genomes present in our database (*P. intermedia*, *P. ruminocola*, *and P. melaninogenica*). During our initial attempts to make taxonomic assignments for cDNA sequences, we used a database that did not contain any *Prevotella* genomes. By adding *Prevotella* genomes, we modestly increased the relative proportion of sequences that could be assigned to a genus (data not shown). The abundance of annotated transcripts from the phylum Bacteroidetes was significantly higher in the FF group. This may represent a higher level of transcriptional activity in FF animals from species in this phylum, as recently reported by Turnbaugh et al. [17]. Alternatively, however, this result may indicate a closer alignment between the genomes of *Bacteroides* species in our database (*B. fragilis NCTC 9343*, *B. fragilis YCH46*, *and B. thetaiotamicron*) and the *Bacteroides* species within the cecal contents of the animals studied.

### Differential gene expression in MF and FF piglets

Gene expression among gut microbes was relatively similar in MF and FF animals, despite taxonomic differences at the level of genus in the observed microbial communities. MG-RAST generated assignments of protein-coding nucleotide sequences to 29 broad functional categories of SEED subsystems and 637 narrow SEED subsystems (Level 1 and Level 3, respectively). In 24 of the 29 major subsystems, the abundance of assigned sequences was not statistically different in the MF and FF groups. Sequences assigned to the Level 1 subsystem of carbohydrates were similarly abundant in the two groups (p = 0.129). Remarkably, the abundance of MF and FF sequences was not significantly different in 94% of Level 3 Subsystems (600 of 637).

Significant differences were observed in five Level 1 SEED subsystems: prophage (p = 0.005), amino acids and derivatives (p = 0.009), potassium metabolism (p = 0.017), respiration (p = 0.018), and motility and chemotaxis (p = 0.04) subsystems. Table 3 and Table S3 list hits to Level 1 and Level 3 SEED Subsystems that were differentially abundant in either the MF group or the FF group; differentially abundant COGs are listed in Table S4. Only two carbohydrate utilization SEED subsystems were differentially represented within the two data sets. Transcripts for utilization of L-arabinose (p = 0.013), a sugar known to accumulate in the distal intestine of FF pigs due to the hydrolysis of plant-based non-starch polysaccharide additives [50], and for utilization of the sugar alcohol mannitol (p = 0.008) were each observed more commonly in the FF group. Two individual COGs, galactose mutarotase (COG2017; p = 0.011) and transketolase (COG0021; p = 0.006) were observed more frequently in the MF group. Galactose mutarotase is an essential enzyme in the metabolism of lactose [51], and transketolase is an enzyme involved in central carbohydrate metabolism that has been shown to link carbon availability with phage resistance, LPS metabolism, and flagellar motor function [52], [53]. Interestingly, in a recent *in vitro* study of *Bifidobacterium* gene expression, transketolase expression was [54] upregulated after exposure to breast milk.

**Table 3:** Differential abundance of gut microbial transcripts assigned to SEED Subsystems.

	Mother-Fed		Formula-Fed		
	Mean relative abundance	Standard error	Mean relative abundance	Standard error	p value
**SEED Level 1 Subsystem**					
Prophage	0.0001	0.0001	0.0004	0.0000	0.005
Amino_Acids_and_Derivatives	0.0741	0.0029	0.0611	0.0022	0.009
Potassium_metabolism	0.0037	0.0004	0.0018	0.0005	0.017
Respiration	0.0361	0.0013	0.0423	0.0018	0.018
Motility_and_Chemotaxis	0.0146	0.0032	0.0065	0.0008	0.040
**SEED Level 3 Subsystem**					
Arginine_Biosynthesis	0.0065	0.0007	0.0020	0.0003	0.001
LOS_core_oligosaccharide_biosynthesis	0.0011	0.0001	0.0003	0.0001	0.003
Aromatic_amino_acid_interconversions_with_aryl_acids	0.0008	0.0002	0.0020	0.0002	0.004
High_affinity_phosphate_transporter	0.0006	0.0000	0.0004	0.0000	0.004
Bacterial_Chemotaxis	0.0057	0.0006	0.0026	0.0005	0.005
Proline__4-hydroxyproline_uptake_and_utilization	0.0116	0.0005	0.0081	0.0007	0.007
Mannitol_Utilization	0.0005	0.0001	0.0012	0.0001	0.008
L-Arabinose_utilization	0.0015	0.0003	0.0034	0.0005	0.013
dTDP-rhamnose_synthesis	0.0015	0.0001	0.0007	0.0002	0.013
Aromatic_Amin_Catabolism	0.0008	0.0002	0.0002	0.0001	0.015
Glycine_and_Serine_Utilization	0.0037	0.0005	0.0056	0.0005	0.016
Methanogenesis	0.0029	0.0009	0.0002	0.0002	0.017
Glycine_cleavage_system	0.0023	0.0005	0.0006	0.0004	0.020
Aromatic_amino_acid_degradation	0.0009	0.0001	0.0003	0.0002	0.020
Alanine_biosynthesis	0.0011	0.0003	0.0003	0.0001	0.024
Purine_Utilization	0.0015	0.0003	0.0048	0.0012	0.026
Ton_and_Tol_transport_systems	0.0095	0.0012	0.0196	0.0034	0.027
Phosphoenolpyruvate_phosphomutase	0.0000	0.0000	0.0006	0.0002	0.028
Terminal_cytochrome_oxidases	0.0006	0.0002	0.0015	0.0002	0.029
Oxidative_stress	0.0267	0.0042	0.0140	0.0021	0.029

Relative abundance of transcripts represents the number of sequences assigned to a subsystem for an individual animal divided by the total number of sequences for that animal. Mean relative abundance represents the average of these values in either the MF or FF treatment groups. A treatment group was considered to be enriched in transcripts assigned to a SEED subsystem if the p value was less than 0.05 for Level 1 subsystems and less than 0.03 for Level 3 subsystem. Subsystems with a mean relative abundance less than 0.0005 in both MF and FF groups were excluded. The table includes only a partial list of differentially abundant subsystems; a complete table is provided in Table S3.

Significant differences between the MF and FF groups were evident in the abundance of transcripts related to amino acid metabolism. The MF group was markedly enriched in sequences encoding for enzymes that contribute to arginine metabolism, e.g. arginine deaminase (COG2235; p = 0.001) and ornithine aminotransferase (COG4992; p = 0.003). In neonates, arginine is synthesized in the gut from proline [55]; we found that transcripts for proline biosynthesis were equally abundant in the two groups (p = 0.56) but transcripts for proline and 4-hydroxyproline utilization were more abundant in the MF group (p = 0.007). The MF group was also enriched in sequences associated with glycine cleavage (p = 0.02), alanine biosynthesis (p = 0.02), and histidine degradation (p = 0.03), whereas the FF group was enriched with sequences associated with glycine and serine utilization (p = 0.016) and aromatic amino acid degradation (p = 0.02).

The MF data set was enriched with sequences assigned to the oxidative stress subsystem (p = 0.029). This may be consistent with long-held claims of the antioxidant properties of maternal milk in human neonates [56], [57]. Additional sequences enriched within the MF data set included transcripts encoding bacterial chemotaxis proteins CheA, CheV, and CheY (p = 0.005), as well as enzymes that contribute to luminal methanogenesis (p = 0.017). In the FF group, transcripts encoding Ton and Tol type receptors, which are important for iron-mediated bacterial virulence in the gut [58], [59], were significantly more abundant than in the MF group (p = 0.020).

## Discussion

It is now well established that gut microbes contribute to mammalian physiology [60], [61]. Pioneering research has clarified the roles of microbes in the gut in normal development and homeostasis [62], and a new generation of investigations seeks to clarify the role of microbes in disease pathogenesis [63]–[65]. Although a subset of diseases can be linked to the presence of a single causative pathogen, there is a growing recognition of the need to study complete or near complete microbial communities [66], [67]. Prior to the advent of next-generation technologies in nucleotide sequencing and mass spectrometry, molecular studies of entire microbial consortia (rather than selected organisms of interest) were not technically feasible. Although bold advances have been made in understanding the metabolic potential of intestinal microbes based upon a massive amount of DNA sequencing of bacterial isolates and in metagenomic studies [9], [11], few functional studies have been performed characterizing community wide gene and protein expression in the gut.

Here we have presented a metatranscriptomic evaluation of intestinal bacteria in 8 neonatal piglets. In this study, we used an RNA pyrosequencing platform to profile gut microbial gene expression in MF and FF piglets without prior knowledge of which organisms were present. To our knowledge, this study represents the largest number of independent samples (8 subjects) used to date for analysis of community wide gene expression in the gut. Several methodologic considerations of this study warrant mention. First, we performed a single step for mRNA enrichment prior to construction of cDNA libraries, and cDNA sequencing results indicated that the degree of enrichment was modest (19.7% of all RNA sequences were non-ribosomal). In the future, this step in sample processing may not be necessary given the volume and length of sequencing reads available with current sequencing platforms. Second, amplification of cDNA sequences was not necessary in this study, although it has been required in prior metatranscriptomic studies due to low yields of RNA [18], [21]. In the piglet, cecal biomass is plentiful and samples can be immediately cryopreserved after collection without exposure to ambient oxygen. As these techniques are inevitably extended to studies of human fecal samples, the need for signal amplification will need to be re-evaluated depending upon RNA yield. Third, we studied the structure of the microbial communities both by sequencing 16S rDNA amplicons and by characterizing unamplified 16S rRNA sequences within the cDNA libraries. In agreement with results from Urich et al. [23], our analyses indicate that taxonomic assignments based upon DNA 16S amplicons correspond well with assignments derived from ribosomal sequences within cDNA libraries. Fourth, gene transcripts were studied without the presence of corresponding metagenomic data sets. The importance of studying paired mRNA and DNA data sets has not yet been clarified and past work in the field has, in fact, demonstrated low levels of homology between metatranscriptomes and metagenomic scaffolds. However, inclusion of whole genome libraries would have likely partially mitigated the large number of unannotated transcripts that we observed in the current study.

A subset of specific transcripts was relatively abundant in all samples studied, indicating that we have begun to define a core neonatal gut microbial metatranscriptome. As expected, a preponderance of mRNA transcripts corresponded to genes related to metabolism of carbohydrates and proteins. These results align well with recent papers that have defined the nature of carbohydrate utilization genes within a core metagenome (9,31,32). Additionally, commonly observed gut microbial transcripts in our study encode for proteins that enable binding to host epithelium, regulate processing of extracellular polysaccharides, and mediate microbial stress response. The validity of our results is supported by a recent proteomic-based study that characterized circulating antibodies against gut bacteria in human subjects [68]. The most commonly observed antibodies were active against GroEL, enolase, and elongation factor Tu, which were each observed frequently in our gene expression study. The significance of how and why antibodies are formed against gut bacterial antigens requires further study.

A primary goal of this study was to identify differences in the gut microbial communities of MF and FF neonatal piglets. MF samples were enriched with 16S sequences from the taxa *Prevotella*, *Oscillibacter*, and *Clostidium*, whereas the FF samples were enriched with sequences from *Bacteroides*, *Parabacteroides*, and *Alistipes*. Because the animals in the MF and FF piglets did not originate in the same litter of piglets, it is possible that observed differences between the two groups reflected a litter effect. However, studies from our laboratories have shown that litter effects on the gut flora of piglets are reproducibly overshadowed by diet effects (Wang et al., submitted).

Profiles of gene expression were similar. The abundance of sequences from more than 90% of SEED subsystems and COG clusters did not differ between MF and FF datasets was not statistically different. These results suggest that sow's milk and artificial formula, although chemically distinct, induce relatively subtle changes in the gut microbial gene expression. However, several important differences were noted in the transcriptomes of the MF and FF animals. Marked differences were noted in the expression of genes involved in amino acid metabolism. Interestingly, we observed a clear abundance of enzymes linked to arginine metabolism in the MF group of animals. This finding may have clinical relevance because several reports have indicated that an abnormally low serum concentration of arginine, a precursor for nitric oxide production, confers an increased risk for NEC [69], [70], and it is widely accepted that the incidence of NEC is lower in low birthweight infants receiving breast milk [7], [8]. Additionally, the MF data sets were significantly enriched in oxidative stress response genes, which is interesting in light of the oft-stated belief that breast milk provides antioxidant protection for newborns. By contrast, the FF data sets were significantly enriched in sequences encoding the Ton and Tol transport proteins, which have been associated with iron-mediated microbial virulence in animal models of infection [58], [59].

Recent advances in completing microbial genomes and completing metagenomic surveys have demonstrated the vast metabolic potential of the bacteria and archaea present within the mammalian intestine. As DNA-based studies continue, parallel studies of gut microbial function will be essential. Functional studies of gene expression, protein expression, and metabolite production will make it possible to define what is “normal” in the field of enteric microbiology. Eventually, continued progress in this area will allow us to better understand the contributions of microbes to diseases such as NEC, Crohn's disease, and obesity.

## Materials and Methods

### Experimental animals

Animals were managed throughout the study in accordance with requirements of the Institutional Animal Care and Use Committee at the University of Illinois (IACUC protocols 08070 and 08015), in accordance with approved NIH guidelines. Vaginally-delivered piglets were allowed to suckle for 48 h postnatal to obtain colostrum. Four piglets (mother fed, MF) from a single litter remained with the sow throughout the study. Four additional piglets (formula fed, FF), from two separate litters, were fed with formula for the remainder of the study. FF piglets were transported to the animal facilities and housed individually in metabolism cages with 12 h light/dark cycle as previously described [71]. Room temperature was maintained at 25°C with supplemental heat was provided through of radiant heaters suspended above the cages to maintain a local temperature between 30 to 32°C. The FF piglets were fed with a cow milk-based sow milk replacer (Advance Baby Pig LiquiWean; Milk Specialties, Dundee, IL). Formula was prepared fresh each morning at a concentration of 18.3% solids and was offered 14-times daily via a pump into a bowl at a rate of 360 mL/kg body weight. Piglets were weighed each morning and monitored three times per day for general health.

### Sample Collection

Samples were collected on postnatal day 21. Piglets were first sedated with an intramuscular injection of Telazol (7 mg/kg body weight; Fort Dodge Animal Health, Fort Dodge, IA) and then euthanized by intracardiac administration of sodium pentobarbital (Fatal Plus: 72 mg/kg body weight; Vortech Pharmaceuticals, Dearborn, MI). The large intestine was excised and separated into cecum and colon at the cecocolic junction. Tissues and luminal contents from cecum were immediately collected into sterile tubes and snap frozen in liquid nitrogen. The samples were stored at −80°C until processing.

### PCR amplification and analysis of 16S rDNA sequences

DNA was isolated from frozen cecal contents according to QIAamp DNA Stool mini Kit (Qiagen, Alameda, CA) with modifications [72]. Variable regions 1–4 of the 16S rRNA gene were amplified using TaKaRa Ex Taq PCR mixture (TAKARA Bio USA, Madison, WI ) and barcoded PCR primers A-788R and B-27F with the structure as follow: A-788R CGTATCGCCTCCCTCGCGCCATCAGGGACTACCAGGGTATCTAA, B-27F CTATGCGCCTTGCCAGCCCGCTCAG-MID-AGAGTTTGATCCTGGCTCAG. The MIDs used in these experiments were: MID4 AGCACTGTAG, MID5 ATCAGACACG, MID6 ATATCGCGAG, MID7 CGTGTCTCTA, MID9 TAGTATCAGC, MID10 TCTCTATGCG, MID11 TGATACGTCT, MID12 TACTGAGCTA. PCR program was set as follows 95°C 10 min and 30 cycles of 95°C 1 min, 50°C 1 min, 72°C 1.5 min followed by 72°C for 10 minutes. PCR products were purified using AMPure Kit (Agencourt Bioscience, Beverly, MA) and sequenced on GS Titanium 70×75 picotiter plate from adaptor B according to the manufacturer's protocols for GS FLX (Roche Applied Science, Indianapolis, IN). The resulting reads were sorted by barcode using SFF software tools, which also trims the barcode sequence in each read after sorting (Roche Applied Science, Indianapolis, IN). Sequences longer then 100bp were taxonomically annotated to the level of genus using the Ribosomal Database Project Classifier tool [30] with a bootstrap cutoff of 50%.

### RNA isolation and cDNA library construction

The frozen ceca were cut in discs ∼0.5–1 cm of thickness using a cold sterile pruner. Residual intestinal tissue was removed with the pruner and a handheld drill (Dremel, Racine, WI). The samples were kept deeply frozen during the procedure. Total microbial RNA isolation was performed using the RiboPure-Bacteria Kit (Ambion, Austin, TX). The MICROBExpress Bacterial mRNA Purification Kit (Ambion) was used to deplete the pool of 16S and 23S rRNA molecules present in the sample. cDNA libraries were constructed according to random primer cDNA synthesis protocol implemented in Double-Stranded cDNA Synthesis Kit (Invitrogen, Carlsbad, CA). Libraries were size selected in 1% low melting point agarose gel. The region of 250–800bp was removed from the gel and purified. Approximately 1µg of randomly-primed, size selected cDNAs were blunt-ended, adaptor ligated and converted to a single-stranded template DNA library using the GS Titanium General Library Prep Kit (Roche Applied Science, Indianapolis, IN). Libraries were prepared using barcode-containing adaptors in place of the standard Titanium adaptors, following Roche's instructions for preparation barcoded adaptors. The barcode sequences used for each library are listed above (MID 1–8). Libraries were quantified using Qubit reagents (Invitrogen, CA) and average fragment sizes were determined by analyzing 1 µl of the sized cDNA samples on the Bioanalyzer (Agilent, CA) using a DNA 7500 chip. The libraries were pooled in equimolar concentration into a single library. Processing for emulsion PCR, titration and sequencing on a GS FLX was done following the manufacturer's protocols (Roche Applied Science) on GS Titanium 70×75 picotiter plate. The resulting library reads were sorted by barcode using SFF software tools (Roche Applied Science).

### Metatranscriptome sequence analysis

A total pool of initial 454 pyrosequencing reads was first screened for length (>100 bp) and rRNA contamination. Replicate reads were not removed, as a preliminary analysis of COG hits and taxonomic assignments indicated that roughly 1% of sequences were perfect replicates and that their presence did not significantly affect the relative abundance of transcripts (data not shown). Sequences were searched against an in-house database of 18,283 annotated long subunit (LSU) and short subunit (SSU) rRNA sequences compiled from the SILVA database [73] and the Ribosomal Database Project (RDP) [74]. Sequences were removed from further analysis based on BLASTN hits to the rRNA database that met the following criteria: (i) e-value<1e-5, and (ii) alignment length ≥40% of the sequence length. Screened cDNA sequences were assigned functional annotations based upon MG-RAST annotations using the SEED database and also upon best-BLASTX-hits to the NCBI COG database (e-values<1e-5). Taxonomic assignments (from phylum to genus) were made according to best-BLASTN-hits (requiring e-values<1e-5) to an in-house database (Table S2) of 1054 completed genomes from NCBI and the J. Craig Venter Institute.

### Statistical analysis

Statistical tests for differentially abundant COG functions, cDNA-based taxonomic annotations, and 16S-based taxonomic annotations between populations (e.g. 21-day MF, 21-day FF) were made using the Metastats methodology with 1000 permutations to compute nonparametric p-values. Thresholds for significance were determined according to the specific comparison. For general COG categories and Level I and II SEED subsystems, the p-value threshold was 0.05. For individual COGs and level III SEED subsystems, the significance thresholds were 0.02 and 0.03, respectively.

## Supporting Information

Figure S1 Principal component analysis of genus-level taxonomic assignments in MF and FF animals. MF animals (n = 4) represented by blue dots; FF animals (n = 4) represented by black dots.
(0.21 MB PDF)

Table S1 Gut microbial community structure in MF and FF piglets. Relative abundance of organisms was determined by amplification and sequencing of 16S rDNA present within microbial DNA isolated from the cecal contents of each animal. Relative abundance of sequences represents the number of sequences assigned to a given taxa for an individual animal divided by the total number of sequences for that animal. Mean relative abundance represents the average of these values in either the MF or FF groups.
(0.03 MB XLS)

Table S2 In-house collection of bacterial genomes used for taxonomic assigment of cDNA sequences.
(0.08 MB XLSX)

Table S3 Complete list of differentially abundant transcripts assigned to SEED subsystems in MF and FF piglets. Relative abundance of transcripts represents the number of sequences assigned to a subsystem for an individual animal divided by the total number of sequences for that animal. Mean relative abundance represents the average of these values in either the MF or FF treatment groups. A treatment group was considered to be enriched in transcripts assigned to a SEED subsystem if the p value was less than 0.05 for Level 1 subsystems and less than 0.03 for Level 3 subsystem. Subsystems with a mean relative abundance less than 0.0005 in both MF and FF groups were excluded.
(0.03 MB XLS)

Table S4 Differentially abundant COG hits in MF and FF piglets. Relative abundance is defined as the number of hits to an individual COG divided by the total number of hits to individual COGs for each individual subject. COGs were excluded if the mean relative abundance in both MF and FF groups was less than 0.0005. A treatment group was considered to be enriched in transcripts from an individual COG if the p value was less than 0.02.
(0.03 MB XLS)
