# Supplementary figures and images for: Gut Microbial Gene Expression in Mother-Fed and Formula-Fed Piglets
Source: PLoS One. 2010 Aug 27;5(8):e12459. doi: 10.1371/journal.pone.0012459 (PMC2929194; doi:10.1371/journal.pone.0012459)

Observations (axes D1 and D2: 55.25 %)

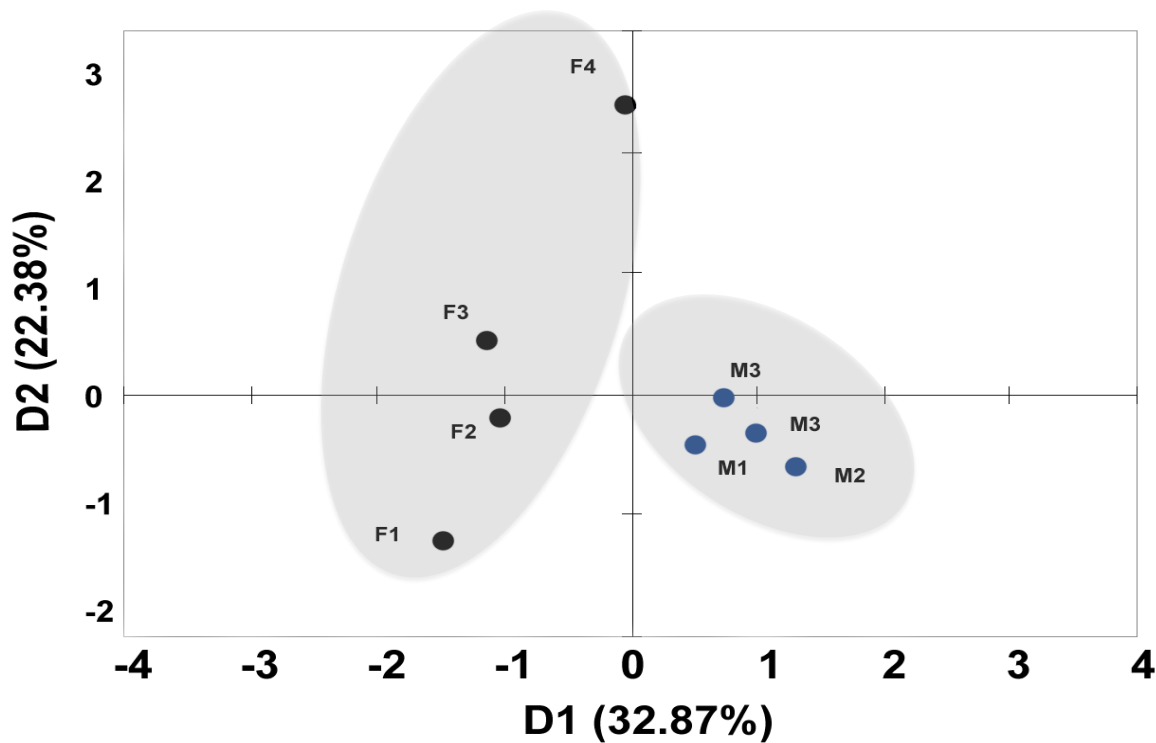

Supplement: Figure S1 — Principal component analysis of genus-level taxonomic assignments in MF and FF animals. MF animals (n = 4) represented by blue dots; FF animals (n = 4) represented by black dots. (0.21 MB PDF) [file pone.0012459.s001.pdf]
